# Modified sports intervention for improving participation goals and activity competence in ambulant children with cerebral palsy: A randomized controlled trial

**DOI:** 10.1111/dmcn.16393

**Published:** 2025-07-03

**Authors:** Ricardo R. Sousa Junior, Georgina L. Clutterbuck, Rafaela F. Guimaraes, Mariane G. Souza, Luana C. Silva, F. Virginia Wright, Ana Cristina R. Camargos, Hércules R. Leite

**Affiliations:** ^1^ Graduate Program in Rehabilitation Sciences, School of Physical Education, Physical Therapy and Occupational Therapy Federal University of Minas Gerais Belo Horizonte MG Brazil; ^2^ School of Health and Rehabilitation Sciences The University of Queensland Brisbane QLD Australia; ^3^ Holland Bloorview Kids Rehabilitation Hospital Toronto ON Canada; ^4^ Department of Physical Therapy University of Toronto Toronto ON Canada; ^5^ CanChild Centre for Childhood‐onset Disability Research McMaster University Hamilton ON Canada

## Abstract

**Aim:**

To evaluate the effectiveness of a modified sports intervention (Sports Stars Brazil) on leisure‐time physical activity participation goals, motor skill performance and capacity, body functions, physical activity levels, physical literacy, and overall participation in ambulant children with cerebral palsy (CP).

**Method:**

In this randomized controlled trial, 38 ambulant children with CP (21 males, 17 females; ages 6–12 years) were randomly assigned to either the intervention or control group. The intervention group participated in eight weekly 1‐hour sessions of modified sports, focused on group‐based motor skill training and introduction to popular Brazilian sports. The control group received their usual physical therapy. Outcomes were assessed at baseline, postintervention, and after 12 weeks. Linear mixed models were used for analysis.

**Results:**

Sports Stars Brazil was more effective than usual care in improving leisure‐time physical activity participation and motor performance, both postintervention and at follow‐up. Groups showed similar outcomes in motor capacity, physical literacy, body functions, physical activity levels, and overall participation immediately postintervention. At 12‐week follow‐up, the Sports Stars group showed greater improvements in motor capacity and moderate to vigorous physical activity.

**Interpretation:**

Sports Stars Brazil is a promising, low‐cost intervention for promoting participation and motor skills in children with CP, particularly in low‐resource settings.

## Graphical abstract

This study evaluates the effectiveness of a modified sports intervention for improving participation goals and activity competence in ambulant children with cerebral palsy.

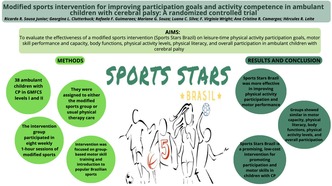

Plain language summary: https://onlinelibrary.wiley.com/doi/10.1111/dmcn.16449

Portuguese translation of this Original Article is available in the online issue.

**Abbreviations**
COPM: Canadian Occupational Performance Measure
TGMD‐2: Test of Gross Motor Development, Second Edition

**What this paper adds**

- Modified sports improved leisure‐time physical activity participation in ambulant children with cerebral palsy.
- Modified sports interventions enhanced motor performance in fundamental motor skills.
- Outcomes in motor skill capacity were comparable to usual physical therapy.
- Similar results in body functions, physical literacy, and overall participation were found.
- Physical activity levels and motor capacity improved at follow‐up in the intervention group.

Ambulant children with cerebral palsy (CP) who are classified in levels I or II of the Gross Motor Function Classification System (GMFCS)^1^ can walk without assistive devices in most environments, but have difficulties with motor skills such as running, jumping, and throwing.^1^ These activity limitations, along with personal and environmental barriers, can lead to less participation in leisure‐time physical activities (e.g. sports or physical recreation), which can result in sedentary behaviours and poor health outcomes over their lifetime.^2^, ^3^ Modified sports interventions might help these children by developing motor skills and promoting future participation in sports or physical recreation.^4^, ^5^, ^6^

Modified sports interventions are grounded in the SPORTS Participation Framework, which outlines a series of stages that children and adolescents with disabilities may move through as they prepare for, enter into, participate in, and excel at sports and physical recreation.^7^ Modified sports interventions represent the ‘P' in the SPORTS acronym (‘Practitioner‐led, peer group sports interventions’).^7^ Modified sports connect health‐related, sports‐focused interventions with mainstream leisure‐time physical activity in the community in the SPORTS Participation Framework's ORTS stages.^7^, ^8^

In recent years, our research group has focused on describing the treatment components and understanding the effectiveness of modified sports interventions for children with neuromotor disabilities such as CP.^7^, ^8^, ^9^, ^10^, ^11^, ^12^, ^13^, ^14^ These interventions typically consist of two main active ingredients: (1) repeated motor skills training and (2) introduction to sports, working through the ‘learning by doing’ mechanism of action.^9^ The main targets are motor skills capacity and performance; however, body functions (e.g. muscle power, balance, and strength) and leisure‐time physical activity participation are indirect outcomes that might also be achieved.^9^ Like other sports‐focused interventions, modified sports interventions also include the achievement of contextual factors such as the physical literacy competencies (e.g. physical, psychological, social, and cognitive components^15^) to effectively achieve lifelong changes in physical activity participation.^8^ Even though ingredients in the physical component are the most reported and clear in modified sports interventions, it is important to integrate ingredient‐related social, cognitive, and psychological competencies, to promote differences in leisure‐time physical activity participation.^8^, ^9^

One example of a modified sports intervention that integrates all physical literacy components is a practitioner‐led, peer‐group programme named Sports Stars.^10^ Its overarching aim is to promote physical activity participation by targeting physical literacy in children with disabilities. The programme focuses on motor skills training, confidence building, teamwork development, and sports education. It was originally created in Australia^10^ and adapted to the Brazilian context (named as Sports Stars Brazil).^12^ The Australian version was well accepted by parents and physical therapists and effective in improving leisure‐time physical activity participation and motor skills.^10^, ^13^ Parents and caregivers in Brazil who were involved in its first roll‐out indicated that the adapted version is a feasible intervention for ambulant children with CP that positively influenced different aspects of all F‐words for child development (Functioning, Family, Fitness, Fun, Friends, and Future).^12^

Although modified sports interventions are likely to improve leisure‐time physical activity participation, motor skills, and body functions in children with CP, the quality of evidence for their effectiveness remains low because of a lack of methodologically strong studies.^9^, ^16^ Randomized controlled trials are needed to strengthen the evidence for already investigated outcomes, such as motor skill gains and physical activity participation. Additionally, because participation is highly influenced by children's environments,^17^ it is crucial to evaluate the effectiveness of interventions in low‐income and middle‐income countries such as Brazil. It is well known that Brazilian physical therapy studies on CP focusing on participation are scarce.^18^ This gap also affects clinical practice, as many Brazilian physical therapists do not offer participation‐focused interventions, instead placing a strong emphasis on body functions and structures.^19^

According to the annual report of the Brazilian Cerebral Palsy Registry, 89.8% of families of people with CP use the Brazilian public health‐care system and 23% do not practise any type of physical activity.^20^ Long waiting lists and lack of specialized physical therapy treatment services are barriers for individuals with CP. Additionally, health care for individuals with CP in Brazil imposes high costs on the Brazilian Unified Health System.^21^ Practitioner‐led, peer‐group interventions such as Sports Stars Brazil may serve as a promising alternative to promote leisure‐time physical activity participation and other important functional outcomes in children with CP.

One of the main objectives of Sports Stars Brazil was to implement a sports‐focused intervention aimed at increasing leisure‐time physical activity participation among Brazilian children with disabilities while contributing to research on modified sports, particularly in the context of low‐income and middle‐income countries.^12^ Further investigation using a randomized controlled trial design would help determine the extent of effectiveness for outcomes that have yet to be explored, such as physical activity levels (including sedentary behaviour and daily physical activity), as well as the social, cognitive, and psychological domains of physical literacy. Moreover, it would provide insights into overall participation across different life contexts, all of which are essential for supporting lifelong engagement in physical activity.

The primary aim of this study was to evaluate the effectiveness of a modified sports intervention (Sports Stars Brazil) for ambulant children with CP, compared with usual physical therapy in terms of attendance (i.e. frequency) and involvement in leisure‐time physical activities (evaluated by parent‐selected goals). Secondary outcomes included motor skill performance and capacity, physical activity levels, physical literacy domains, body functions, and overall participation.

## METHOD

### Study design

This study was a single‐blind, two group, randomized controlled trial (Sports Stars Brazil vs. usual physical therapy control group) with concealed allocation, assessor blinding, and intent‐to‐treat analysis. The study was prospectively registered in the Brazilian Clinical Trials Registry (RBR‐3RWTYW, World Health Organization Universal Trial Number U1111‐1256‐4998) and approved by the ethics review board of Universidade Federal de Minas Gerais, Brazil (CAEE 33238520.5.0000.5149). Written informed consent was obtained from all parents and children before the beginning of the study procedures. The study protocol, describing the methods of this present study with details, can be found elsewhere.^22^ This study was conducted between November 2021 and March 2024.

### Setting

This study was conducted in the multi‐sports courts and running tracks of the School of Physical Education, Physical and Occupational Therapy of the Universidade Federal de Minas Gerais, Minas Gerais, Brazil.

### Study sample, recruiting, and sample estimation

Participants were recruited by convenience from public or philanthropic physical therapy institutions and private clinics in Belo Horizonte, Brazil, and surrounding areas. Children were eligible for the study if they were 6 to 12 years of age at the beginning of the intervention, with a diagnosis of CP, and classified in GMFCS level I or II. They had to be able to understand simple instructions and not have any orthopaedic or cardiorespiratory condition that would prevent them participating in physical activities with peers safely. Because children randomized to the Sports Stars Brazil group would be temporarily suspended from their usual physical therapy treatment (1–2 hours of individual therapy per week), parents and therapists first needed to agree that they might benefit from the programme and did not have other therapy goals unrelated to the study (e.g. upper extremity function, alignment) that would make it harmful for them to discontinue regular physical therapy. Those not able to suspend physical therapy treatment were not considered eligible for study enrolment. The target sample size of 38 children was based on the original Sports Stars study,^13^ where an effect size of 1.05 was found in the difference in means of the participation outcome (i.e. parent‐set frequency and/or attendance participation goals measured by a modified administration of the Canadian Occupational Performance Measure, COPM)^23^ in comparison with the usual care, posttreatment.^10^ Software GPower 3.1.9 (Heinrich Heine University, Düsseldorf, Germany) was used for sample size calculation, considering a power of 80%, *α* = 5%, and 20% study attrition.

### Randomization and blinding

Children were recruited and enrolled by the main interventionist (RRSJ). Children were randomized into the Sports Stars Brazil group or usual physical therapy control group by one investigator (HRL). A random number generator created a random sequence of numbers which were concealed in individual numbered opaque sealed envelopes. This sequence randomized children into the Sports Stars Brazil group (concealed even numbers) or usual physical therapy control group (concealed odd numbers). Randomization ran in stages; each stage being completed when a subgroup of 8 to 16 participants was recruited (to complete a group of four to eight children in the intervention group). A new sequence was generated for each subgroup randomization until 38 children were allocated. Owing to the small number of available possible participants willing to participate in the study, randomization was only based on the random number generator and not on participants' characteristics (e.g. GMFCS level). The assessors were blinded to group allocation.

### Intervention groups

#### Sports Stars Brazil intervention

**FIGURE 1:**
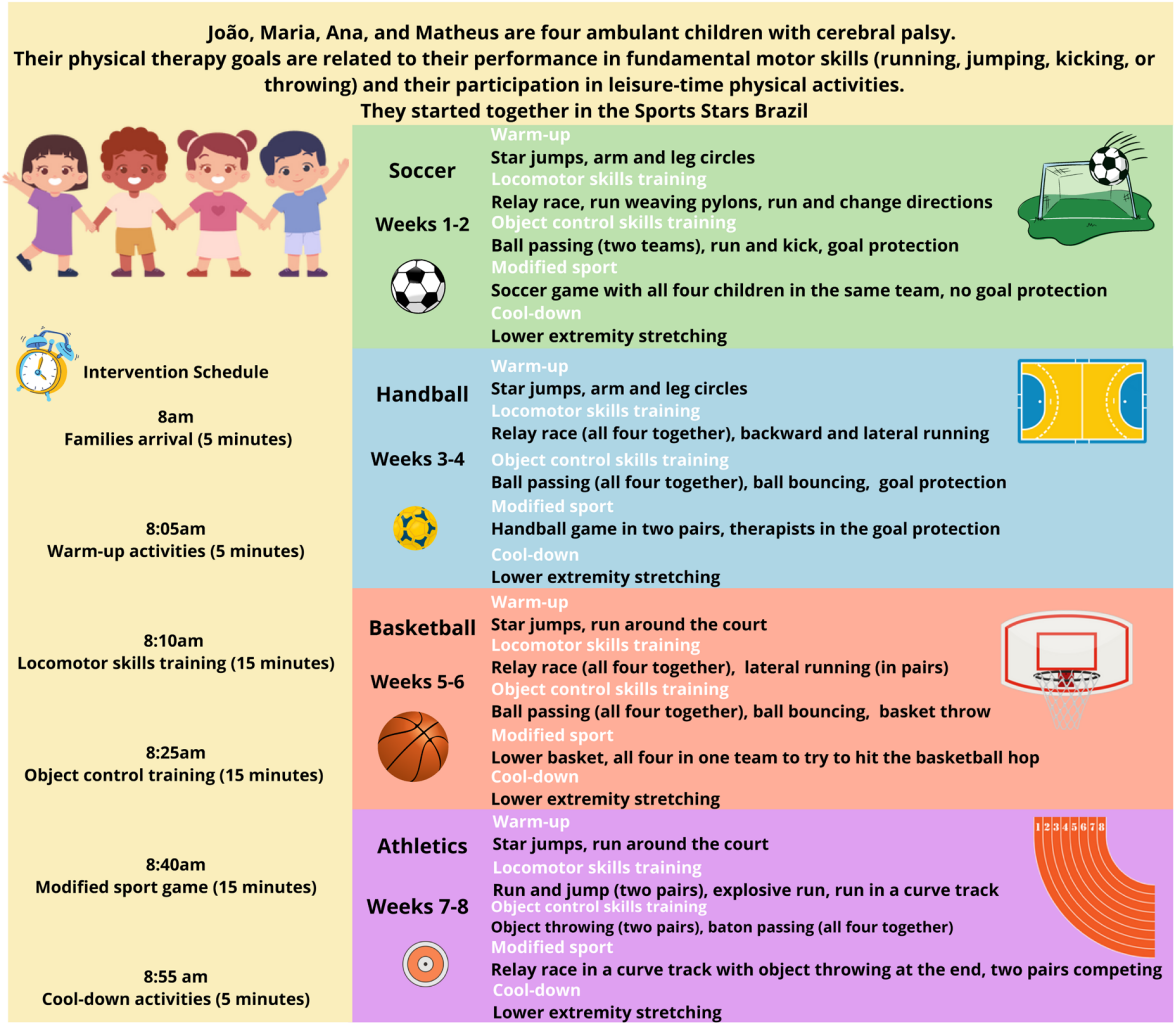
Session sample of Sports Stars Brazil.

This practitioner‐led, peer‐group, modified sports intervention contained four to eight participants in each programme group. It was led by one trained physical therapist together with physical education professionals and approximately four to six graduate students of both disciplines. Children in Sports Stars Brazil attended eight 1‐hour sessions once per week, with training focused on four different sports (2 weeks each): soccer, handball, basketball, and athletics. Modified sports interventions present varied dosage parameters;^9^ for this study, we opted to use the same dosage and follow‐up periods as Clutterbuck et al. in the Sports Stars Australia study, because that showed positive results on children's participation, and to allow comparative discussion among both Sports Stars versions.^13^ This low dosage might also be suitable for application in low‐income and middle‐income countries such as Brazil. The main active ingredients of this intervention were the repetitive active practice of sports‐related motor skills and the introduction to the target sports (soccer, handball, basketball, and athletics), with modification of their complexity (i.e. changing rules, number of participants, or equipment to introduce the sport). Motor skills training was conducted by providing opportunities for structure and repeated practice, using progressive graduation of task complexity, verbal cues, feedback strategies, and motivational strategies to build cognitive, social, and psychological components of physical literacy. The main interventionist (RRSJ) was a paediatric physical therapist with 9 years of experience with children with CP and was trained by Sports Stars' original developer (GLC) in four online meetings; the local interventionist then trained the other members of the team before the start of the study. Details of Sports Stars Brazil sessions and case examples can be found in the published protocol of the study^22^ and in a previous study.^12^ A session sample is also provided in Figure 1.

#### Usual physical therapy

Participants in the control group received their usual physical therapy care. In Brazil, this usually includes interventions such as gross motor training, muscle strengthening, and balance training.^19^ We intended to collect information about participants' usual physical therapy (e.g. therapy goals, intervention characteristics treatment plan, dosage, and adherence) by directly contacting children's physical therapists. However, therapists did not collaborate with usual care information. Hence, information on the usual physical therapy group relied on parents' and caregivers' recalls, as described in Table S1. According to parents and caregivers, these interventions primarily focused on improving children's balance, coordination, and alignment while performing fundamental skills (e.g. walking, running, and object control) in a clinical setting. The sessions included exercises targeting strength, balance, mobility, and coordination, as well as treadmill training. During the study all children received one or two individual physical therapy sessions per week (mostly 45 minutes each), primarily in public institutions. This group received a total of 12 to 24 hours of therapy, for 8 weeks. After completion of their 12‐week follow‐up assessment, children in this control group were invited to receive the 8‐week modified sports intervention, after the study completion, only for ethical reasons.

### Baseline sample characteristics

Children's age, sex, type of CP (spastic, dyskinetic, ataxic, or mixed), distribution (unilateral or bilateral), GMFCS^1^ level, and manual function classification (Manual Ability Classification System)^24^ were collected by the investigators on entry into the study. We also collected the frequency (days per week) of leisure‐time physical activity participation, including physical education in school and community physical activities. In addition, the Community Environment section of the Participation and Environmental Measure for Children and Youth was used to characterize participants' environments. This questionnaire provides the percentage of environmental support and barriers according to whether features such as time, resources, or money could hinder children's participation.^25^

### Outcome measures

Table 1 outlines the outcome measures used. Further details of these measures and administration procedures can be found in the published study protocol.^22^ Data collection occurred at baseline, postintervention, and 12‐week follow‐up, as also conducted by the original Sports Stars study.

**TABLE 1:** Primary and secondary outcome measures.

Primary outcome			
Outcome area		Instrument/questionnaire	Description and procedures
Attendance (i.e. frequency of attending, or the range or diversity of activities^17^) and involvement (i.e. engagement, motivation, and confidence, or social connection^17^) in leisure‐time physical activity participation		COPM	The COPM is a person‐centred outcome measure. In this semi‐structured questionnaire, the child and/or their family rank and grade goals for performance and satisfaction on a 10‐point scale.^23^ For this study, a modified administration was conducted, as Clutterbuck et al.,^11^ where parents or caregivers chose two goals in the COPM's leisure domain: one for attendance (e.g. ‘To attend in physical education activities at school twice in a week’ or ‘Spending more time during the day practicing jumping and running games’) and one for involvement in a leisure‐based physical activity goal (e.g. ‘To be confident and engaged when participating at running activities with friends’ or ‘Getting less frustrated when making mistakes or losing during games with friends’).
Secondary outcomes			
Outcome area and measure		Instrument/questionnaire	Description and procedures
Motor skills	Performance	COPM	A third goal in the modified COPM administration set by parents/caregivers related to children's performance on motor skills (e.g. running, jumping, kicking, or throwing).
	Capacity	TGMD‐2 and Challenge Test	The TGMD‐2 evaluates movement patterns, when executing fundamental motor skills.^26^ The Challenge Test measures 25 items with advanced motor skills (e.g. accuracy and speed during running, jumping, or throwing) capacity in ambulant children with CP.^27^
Body functions	Muscle power/agility	MPST and 10X5ST	The 10X5ST is an agility and anaerobic capacity measure for ambulant individuals with CP. The MPST measures power capability.^28^
	Balance	Kids‐Mini‐BESTest	The Kids‐Mini‐BESTest is a standardized test for balance that has 16 items of anticipatory postural adjustments, reactive postural responses, sensory orientation, and stability in gait.^29^
Physical activity levels	Percentage of sedentary behaviour	Accelerometer (ActiGraph wGT3X‐BT, ActiGraph, Pensacola, FL, USA)	This is a small, non‐invasive device that captures the magnitude of trunk acceleration in three planes.^30^, ^31^ Participants use the device on an elastic belt situated above the dominant hip. At the three time‐points of this study, ActiGraph devices were worn by the participants during 7 consecutive days (5 weekdays and 2 weekend days). The device was used during usual waking hours and removed during sleep and water activities. Periods of non‐wear (maximum of 2 consecutive minutes of zero counts) were automatically detected by the device (Troiano algorithm^31^). Devices were returned after day 7 in each wear cycle for data extraction on the ActiLife 6 software (ActiGraph, LLC, Pensacola, Florida, USA).
	Percentage of LPA		
	Percentage of MVPA		
	Mean time per day in MVPA		
Physical literacy profile across physical, social, psychological, and cognitive domains		PLP‐Quest	PLP‐Quest is a new, validated tool that assesses physical, social, cognitive, and psychological domains and its components, according to parents' perceptions of their child's physical literacy. It has 22 items scored on a 3‐point extent of performance scale.^32^
Overall life participation		PEM‐CY	The PEM‐CY is a parent‐report questionnaire that measures the frequency, involvement, and desire for change in participation in three different settings: home, school, and community.^25^

Abbreviations: 10X5ST, 10 metres per 5 seconds sprint test; CI, confidence intervals; COPM, Canadian Occupational Performance Measure; Kids‐Mini‐BESTest, Kids Mini Balance Evaluation Systems Test; LPA, light physical activity, MVPA, moderate to vigorous physical activity; MPST, Muscle Power Sprint Test, PEM‐CY, Participation and Environment Measure for Children and Youth; PLP‐Quest, Physical Literacy Profile‐Questionnaire; SD, standard deviation; TGMD‐2, Test of Gross Motor Development, Second Edition.

### Procedures

Outcome measures were administered by two blinded physical therapists formally trained by senior researchers. Physical tests were divided between two sessions within a week: (1) Test of Gross Motor Development, Second Edition (TGMD‐2), Muscle Power Sprint Test, 10 metres per 5 seconds sprint test, and Kids Mini Balance Evaluation Systems Test (Kids‐Mini‐BESTest) (administered by evaluator 1); or (2) Challenge Test (administered by evaluator 2) to avoid over‐fatiguing the children. Parents/caregivers responded to the questionnaires online (except for the COPM). Assistance was given by the evaluators in case of difficulties.

Parents/caregivers were invited to observe all physical assessments of their children, and then the COPM was conducted in person by evaluator 1. At this time, the examiner asked parents and caregivers to choose the participation goals, giving examples and helping them to structure the goals. Children were encouraged to participate in this process together with their families, by expressing their preferences.

Participants wore ActiGraph devices for 7 consecutive days (5 weekdays and 2 weekend days) preceding the assessments. The device was used during usual waking hours and removed during sleep and water activities. Periods of non‐wear (a maximum of 2 consecutive minutes of zero counts) were automatically detected by the device (Troiano algorithm).^30^ Devices were returned after day 7 in each wear cycle for data extraction on the ActiLife 6 software (ActiGraph, Pensacola, FL, USA). All assessments and goal setting were conducted before randomization. Adherence to the Sports Stars Brazil intervention and all adverse effects were documented by the main interventionist.

### Adherence to the study's published protocol

This study was conducted according to the published protocol,^22^ with minor exceptions as follows. Regarding recruitment, our original protocol also included adolescents; however, the actual study was limited to children aged 6 to 12 years owing to difficulties in recruiting adolescents with CP in our region. A pilot study on adolescents with CP is underway. Regarding outcome measurements, the Physical Literacy Profile‐Questionnaire was identified by its initial name in our protocol paper (‘Parent/caregivers’ perceptions across physical literacy domains') because it was being developed at the time of the protocol's publication. We were not able to collect children's attendance (mean minutes per day) in sports and physical recreation activities as planned because parents did not adhere to completing the daily logs. To evaluate physical activity levels using accelerometery, we opted to replace the overall cut‐off points mentioned in our study protocol^22^ for those described by Trost et al.^30^ because they are specific for children in GMFCS levels I or II. Lastly, we planned to evaluate COPM proportions of participants who achieved the minimal clinically important difference values for the COPM scores, considering a change higher than 2 points in COPM as clinically important. Because current evidence has refuted the minimal clinically important difference values for the COPM,^33^ we decided not to proceed with this analysis.

### Statistical analysis

Descriptive statistics summarized participants' characteristics (age, sex, and CP classification, sociodemographic characteristics). Data were reported using mean and standard deviation. Normality of the outcomes data was investigated using the Shapiro–Wilk test. Baseline differences between groups were assessed using an independent *t*‐test or Mann–Whitney *U* test. Linear mixed models^34^ were used to analyse the primary and secondary outcomes, comparing the Sports Stars Brazil group and the usual physical therapy group after the intervention and at follow‐up. The models included random intercepts and fixed coefficients, incorporating treatment groups, time points (baseline, postintervention, and follow‐up), and treatment × time interactions. Results were reported as mean differences with 95% confidence intervals (CIs) and Cohen's effect sizes (*d*). Effects sizes were interpreted considering Cohen's criteria: small effects (<0.14), moderate effects (0.30–0.60), and large effects (>0.60).^35^ All statistics were conducted in the Statistical Package for the Social Sciences (SPSS) software (version 26, IBM Corp., Armonk, NY, USA).

## RESULTS

Thirty‐eight children were recruited and evaluated at baseline. Participants' demographic characteristics are presented in Table 2. The groups were similar in their demographic and environmental characteristics, frequency of physical activity participation, and for all primary and secondary outcome measures at baseline. There were no dropouts after randomization or during the study in either group. However, there were three to seven cases with missing data in some postintervention and follow‐up assessments (see CONSORT flow diagram in Figure S1 with numbers and main reasons).

**TABLE 2:** Sample characteristics.

Sample characteristics	Usual physical therapy (*n* = 19)	Sports Stars Brazil (*n* = 19)
Mean age, years:months	8:4 (SD 1:11, minimum 8:0, maximum 11:0)	8:11 (SD 1:11, minimum 6:0, maximum 12:0)
Type of CP	Spastic: 18 (94%) Ataxic: 1 (6%) Mixed:0 (0%)	Spastic: 18 (94%) Ataxic: 0 (0%) Mixed: 1 (6%)
Biological sex	Male: 10 (53%) Female: 9 (47%)	Male: 11 (57%) Female: 8 (43%)
Type of CP	Unilateral: 12 (63%) Bilateral: 7 (34%)	Unilateral: 9 (47%) Bilateral: 10 (53%)
GMFCS level	I: 13 (68%) II: 6 (32%)	I: 13 (68%) II: 6 (32%)
MACS level	I: 13 (68%) II: 4 (21%) III: 2 (11%)	I: 12 (63%) II: 6 (32%) III: 1 (5%)
Baseline frequency of physical activity per week	0 days: 17 (89.48%) 1 day: 2 (10.52%) 2 days: 0	0 days: 16 (84.21%) 1 day: 1 (5.3%) 2 days: 2 (10.52%)
Baseline community environment (measured by PEM‐CY)	Usual physical therapy (*n* = 19)	Sports Stars Brazil (*n* = 19)
Supports	60.57% (SD 6.19)	60.94% (SD 5.21)
Barriers	39.25% (SD 6.20)	38.84% (SD 5.22)
Facilitators	86.10% (SD 3.55)	89.00% (SD 2.32)
Resources	78.42% (SD 2.85)	78.84% (SD 3.37)
General support	82.84% (SD 2.72)	82.10% (SD 2.50)

Abbreviations: CP, cerebral palsy; GMFCS, Gross Motor Function Classification System; MACS, Manual Ability Classification System; PEM‐CY, Participation and Environment Measure for Children and Youth; SD, standard deviation.

### Adherence and adverse effects

The mean attendance for the Sports Stars Brazil group was 6.68 sessions (SD 1.29) of the eight sessions (83.5%) over the 8 weeks. Only one participant in this group reported a minor adverse event, presenting with calf postexercise muscle pain after the first day of intervention which resolved 1 day after and did not recur. The usual physical therapy group attended one or two physical therapy sessions per week over the 8 weeks (mean 2 days per week, SD 0.75). Even though we were unable to collect actual data on adherence (percentage of attended sessions) to usual physical therapy treatment from the perspective of children's physical therapists, parents reported that children had few absences during the study period.

### Primary outcomes

Table 3 presents the list of all COPM goals, chosen by parents and caregivers at baseline. These goals, set by parents, in the intervention and usual physical therapy groups presented similar content.

**TABLE 3:** Participation (attendance/involvement in physical activities) and motor performance goals set by parents and caregivers.

Attendance in leisure‐time physical activity	Involvement in leisure‐time physical activity	Performance in fundamental gross motor skills
**Sports Stars Brazil group** ‘Playing tag more often with friends at home’ ‘Playing soccer games with cousins more often’ ‘Spending more time practicing running’ ‘Starting to practice swimming at least 2 times a week’ ‘Starting to practice favorite sports (basketball, gymnastics) 1 to 2 times a week’ ‘Spending more time during the day practicing jumping and running games’ ‘Starting to practice a sport (e.g. swimming) at least once a week’ (two children) ‘Playing ball games more often’ (two children) ‘Participating more in physical education at school’ (three children) ‘Playing soccer longer with friends’ (three children) ‘Starting to practice a sport at least one in a week’ (three children) **Usual physical therapy group** ‘Spending more time during the day practicing jumping and running games’ ‘Playing ball activities with their sister more often during the week’ ‘Playing on the trampoline more often during the week’ ‘Starting to play soccer with friends’ (two children) ‘Playing soccer longer with friends’ (two children) ‘Participating more in physical education at school’ (four children) ‘Starting to practice swimming at least once in a week’ (three children) ‘Starting to practice a sport at least 2 times a week’ (three children) ‘Starting to practice a sport at least once a week’ (two children)	**Sports Stars Brazil group** ‘Being more motivated to play soccer with cousins’ ‘Being more confident in activities involving balance, such as running and jumping’ ‘Being more involved in games at the park on weekends’ ‘Having more security and confidence to practice swimming’ ‘Having more confidence to change direction while riding a bike with parents’ ‘Feeling more secure and confident to play with classmates’ ‘Being more focused during soccer games’ (two children) ‘Knowing how to handle defeats (get confident and less frustrated) while playing with friends’ (two children) ‘Feeling more confident to participate in games with other children’ (three children) ‘Having more focus during games with friends’ (three children) ‘Being more engaged while participating in physical activities’ (three children) **Usual physical therapy group** ‘Having more confidence to play soccer with other children’ ‘Feeling less afraid to participate in ball activities’ ‘Getting less frustrated when making mistakes or losing during games with friends’ ‘Having more security and confidence to play with unfamiliar children’ ‘Being less afraid of falling while running and playing with other children’ ‘Feeling more confident to participate in games with other children’ (two children) ‘Feeling more secure and confident to play with classmates’ (two children) ‘Feeling more confident to participate in physical activities at school’ (two children) ‘Having more focus during games with friends’ (three children) ‘Being more engaged while participating in physical activities’ (five children)	**Sports Stars Brazil group** ‘Jumping higher’ ‘Running with more coordination’ ‘Catching small balls’ ‘Being able to bounce a basketball with both hands’ ‘Throwing balls with both hands’ ‘Catching the ball in motion’ ‘Bouncing a basketball’ (two children) ‘Kicking a ball’ (two children) ‘Being able to throw a ball’ (three children) ‘Running and kicking the ball nonstop' (three children) ‘Running with more balance’ (three children) **Usual physical therapy group** ‘Running with better quality’ ‘Kicking with the affected leg’ ‘Jumping with the affected foot’ ‘Running and kicking the ball nonstop' ‘Kicking a soccer ball with direction’ ‘Throwing a ball with both hands’ ‘Being able to hop on one foot with more balance’ ‘Running and kicking the ball nonstop' (two children) ‘Being able to throw a ball’ (three children) ‘Running with more coordination’ (three children) ‘Kicking a ball’ (four children)

Table 4 displays the results of the primary outcomes comparing both groups. Immediately after 8 weeks of intervention, Sports Stars Brazil was more effective than usual physical therapy in improving goals related to leisure‐time physical activity attendance (COPM performance: mean difference = 2.05 [0.42–3.68], *d* = 0.97; COPM satisfaction: mean difference = 2.05 [0.23–3.87], *d* = 0.80) and involvement (COPM performance: mean difference = 1.84 [0.67–3.01], *d* = 0.65; COPM satisfaction: mean difference = 2.10 [0.51–3.69], *d* = 1.01). The magnitude of differences between Sports Stars Brazil and usual physical therapy in the leisure‐time physical activity goal improvements was generally high. Superiority of Sports Stars Brazil was maintained at 12‐week follow‐up, only for the attendance goal (COPM performance: mean difference = 1.85 [0.11–3.58], *d* = 0.80; COPM satisfaction: mean difference = 1.72 [−0.21 to 3.67], *d* = 0.65), although with smaller effect sizes. Within‐group changes over the time data are presented in Table S2.

**TABLE 4:** Sports Stars Brazil (*n* = 19) and usual physical therapy (*n* = 19) between‐group comparisons for COPM total scores.

Participation goals measured by COPM (unit: score points 1–10)	Time point	Sports Stars Brazil, mean (SD)	Usual physical therapy, mean (SD)	Mean difference between groups (95% CI)	Effect size
Attendance: performance	Baseline	3.00 (2.47)	2.33 (1.97)	—	—
	Postintervention	5.77 (3.42)	3.05 (2.53)	2.05 (0.42 to 3.68)	0.97
	12‐week follow‐up	6.33 (3.43)	3.73 (2.98)	1.85 (0.11 to 3.58)	0.80
Attendance: satisfaction	Baseline	3.16 (3.05)	2.55 (2.40)	—	—
	Postintervention	6.16 (3.71)	3.50 (3.24)	2.05 (0.23 to 3.87)	0.85
	12‐week follow‐up	6.60 (3.52)	4.26 (3.59)	1.72 (−0.21 to 3.67)	0.65
Involvement: performance	Baseline	4.94 (1.80)	5.31 (1.85)	—	—
	Postintervention	7.15 (2.33)	5.68 (2.18)	1.84 (0.67 to 3.01)	0.65
	12‐week follow‐up	7.25 (2.48)	6.75 (1.73)	0.86 (−0.38 to 2.10)	0.23
Involvement: satisfaction	Baseline	4.94 (2.50)	4.52 (2.29)	—	—
	Postintervention	7.73 (2.28)	5.21 (2.67)	2.10 (0.51 to 3.69)	1.01
	12‐week follow‐up	8.00 (2.25)	6.43 (2.36)	1.04 (−0.63 to 2.72)	0.68

Abbreviations: CI, confidence intervals; COPM, Canadian Occupational Performance Measure; SD, standard deviation.

### Secondary outcomes

Table 5 presents the results for the secondary outcomes. Sports Stars Brazil was more effective than usual physical therapy in improving goals related to motor performance (COPM performance: mean difference = 2.21 [1.23–3.18], *d* = 0.80; COPM satisfaction: mean difference = 2.47 [1.27–3.66], *d* = 0.96). The magnitude of the difference between Sports Stars Brazil and usual physical therapy in motor performance goal improvements was high. These effects were sustained at the 12‐week follow‐up (COPM performance: mean difference = 2.17 [1.14–3.21], *d* = 0.67; COPM satisfaction: mean difference = 2.14 [0.87–3.41], *d* = 0.89), with moderate to high effect sizes. In addition, Sports Stars Brazil outperformed usual physical therapy in motor skills capacity (TGMD‐2 object control: mean difference = 6.80 [1.82–11.78], *d* = 0.64; TGMD‐2 overall: mean difference = 9.00 [0.61–17.40], *d* = 0.51) and in the percentage of time spent in moderate to vigorous physical activity at 12‐week follow‐up (mean difference = 2.89 [0.33–5.46], *d* = 0.34), with moderate effect sizes. Negligible differences with small effect sizes were observed in the remaining outcomes. Within‐group changes over the time data are presented in Table S2.

**TABLE 5:** Comparison of scores for Sports Stars Brazil (*n* = 19) and usual physical therapy (*n* = 19) groups: between‐group analysis of secondary outcome measures.

	Time point	Sports Stars Brazil, mean (SD)	Usual physical therapy, mean (SD)	Mean difference between groups (95% CI)	Effect size
Motor performance goal: performance Measured by COPM Unit: score points 1–10	Baseline	4.57 (1.74)	5.26 (1.66)	—	—
	Postintervention	7.21 (1.96)	5.68 (1.84)	2.21 (1.23 to 3.18)	0.80
	12‐week follow‐up	7.71 (1.82)	6.06 (1.87)	2.17 (1.14 to 3.21)	0.67
Motor performance goal: satisfaction Measured by COPM Unit: score points 1–10	Baseline	5.47 (2.16)	5.68 (2.23)	—	—
	Postintervention	8.31 (2.10)	6.05 (2.57)	2.47 (1.27 to 3.66)	0.96
	12‐week follow‐up	8.56 (1.89)	6.50 (2.65)	2.14 (0.87 to 3.41)	0.89
Motor skills locomotor capacity Measured by TGMD‐2 Unit: score points 0–48	Baseline	29.15 (10.43)	31.22 (9.34)	—	—
	Postintervention	32.33 (9.57)	37.38 (9.81)	—2.06 (−7.04 to 8.78)	0.20
	12‐week follow‐up	34.66 (9.16)	32.16 (9.90)	2.44 (−2.56 to 5.23)	0.22
Motor skills object control capacity Measured by TGMD‐2 Unit: score points 0–48	Baseline	28.40 (10.67)	31.23 (8.73)	—	—
	Postintervention	34.55 (7.42)	33.23 (6.98)	4.29 (−0.47 to 9.07)	0.18
	12‐week follow‐up	38.00 (5.73)	33.46 (8.15)	6.80 (1.82 to 11.78)	0.64
Motor skills overall capacity Measured by TGMD‐2 Unit: score points 0–96	Baseline	57.55 (18.72)	65.05 (17.15)	—	—
	Postintervention	66.88 (15.86)	70.61 (16.00)	2.08 (−5.83 to 10.00)	0.23
	12‐week follow‐up	72.92 (13.68)	65.08 (16.53)	9.00 (0.61 to 17.40)	0.51
Motor skills capacity Measured by Challenge Test Unit: score points as percentage	Baseline	35.84 (20.10)	40.55 (21.23)	—	—
	Postintervention	41.40 (21.57)	47.57 (24.67)	—3.32 (−8.04 to 1.39)	0.26
	12‐week follow‐up	42.72 (19.10)	42.43 (24.23)	0.52 (−5.42 to 4.36)	0.01
Physical activity levels: sedentary behaviour Measured by accelerometer Unit: time as percentage	Baseline	59.45 (13.73)	54.48 (9.94)	—	—
	Postintervention	57.29 (11.86)	57.01 (8.60)	—2.05 (−6.97 to 2.87)	0.02
	12‐week follow‐up	55.52 (10.97)	55.73 (10.45)	—3.79 (−8.66 to 1.06)	0.01
Physical activity levels: time in LPA Measured by accelerometer Unit: time as percentage	Baseline	30.11 (10.01)	33.62 (5.70)	—	—
	Postintervention	31.52 (8.09)	32.07 (5.06)	1.25 (−2.00 to 4.52)	0.08
	12‐week follow‐up	32.19 (7.72)	33.34 (8.06)	1.29 (−1.92 to 4.52)	0.14
Physical activity levels: time in MVPA Measured by accelerometer Unit: time as percentage	Baseline	10.37 (4.35)	11.87 (3.46)	—	—
	Postintervention	11.17 (4.58)	10.86 (4.11)	1.00 (−1.59 to 3.60)	0.07
	12‐week follow‐up	12.18 (4.78)	10.53 (4.88)	2.89 (0.33 to 5.46)	0.34
Physical activity levels: mean MVPA per week Measured by accelerometer Unit: time in minutes	Baseline	65.63 (28.68)	69.05 (20.64)	—	—
	Postintervention	64.29 (32.81)	61.53 (21.89)	2.51 (13.69 to 18.72)	0.09
	12‐week follow‐up	68.56 (31.32)	51.76 (20.64)	15.59 (−0.42 to 31.60)	0.63
Physical literacy Measured by PLP‐Quest Unit: score points as percentage	Baseline	69.94 (15.79)	70.45 (17.29)	—	—
	Postintervention	81.69 (15.02)	75.43 (15.98)	6.76 (−3.35 to 16.88)	0.40
	12‐week follow‐up	79.05 (19.12)	72.05 (20.99)	6.56 (−4.43 to 17.00)	0.34
Balance Measured by Kids‐Mini‐BESTest Unit: score points 0–34	Baseline	26.25 (4.20)	27.52 (2.83)	—	—
	Postintervention	25.56 (6.98)	28.38 (2.46)	—1.05 (−3.39 to 1.29)	0.53
	12‐week follow‐up	26.73 (3.57)	27.23 (3.39)	0.02 (−2.34 to 2.39)	0.14
Muscle power: mean Measured by MPST Unit: power (watts)	Baseline	69.02 (39.56)	77.40 (49.78)	—	—
	Postintervention	75.23 (41.96)	79.19 (66.57)	3.05 (−10.91 to 17.02)	0.07
	12‐week follow‐up	73.41 (40.56)	56.19 (42.43)	12.67 (−1.46 to 26.80)	0.41
Muscle power: peak Measured by MPST Unit: power (watts)	Baseline	81.55 (47.61)	95.32 (64.17)	—	—
	Postintervention	93.33 (51.68)	94.09 (72.84)	10.75 (−6.05 to 27.56)	0.01
	12‐week follow‐up	91.36 (44.62)	79.72 (59.98)	10.65 (−6.36 to 27.66)	0.22
Agility Measured by 10X5ST Unit: seconds	Baseline	36.49 (12.51)	36.03 (11.95)	—	—
	Postintervention	39.16 (19.79)	35.26 (8.68)	0.85 (−3.19 to 4.89)	0.25
	12‐week follow‐up	35.37 (6.26)	37.20 (7.95)	1.16 (−5.33 to 3.00)	0.25
Overall participation: school frequency Measured by PEM‐CY Unit: score points 0–7	Baseline	5.36 (1.53)	5.15 (1.80)	—	—
	Postintervention	5.26 (1.28)	4.84 (1.64)	0.21 (−0.70 to 1.12)	0.28
	12‐week follow‐up	5.33 (1.23)	5.26 (1.38)	0.10 (−1.10 to 0.88)	0.05
Overall participation: school involvement Measured by PEM‐CY Unit: score points 0–5	Baseline	4.42 (0.76)	3.89 (1.24)	—	—
	Postintervention	4.47 (0.61)	4.15 (1.21)	−0.21 (−0.73 to 0.30)	0.33
	12‐week follow‐up	4.60 (0.73)	4.33 (0.89)	−0.19 (−0.75 to 0.37)	0.33
Overall participation: school desire for change Measured by PEM‐CY Unit: percentage	Baseline	64.21 (34.19)	71.57 (37.03)	—	—
	Postintervention	55.78 (35.63)	63.15 (34.80)	0.00 (−20.74 to 20.74)	0.20
	12‐week follow‐up	54.66 (34.19)	54.66 (26.69)	6.85 (−15.66 to 29.39)	0.01
Overall participation: school number of activities Measured by PEM‐CY Unit: percentage	Baseline	3.57 (1.12)	3.00 (1.41)	—	—
	Postintervention	3.94 (1.12)	3.52 (1.42)	−0.15 (−1.00 to 0.68)	0.44
	12‐week follow‐up	3.60 (1.24)	3.60 (1.40)	−0.53 (−1.45 to 0.38)	0.01
Overall participation: community frequency Measured by PEM‐CY Unit: score points 0–7	Baseline	4.42 (1.16)	4.42 (1.26)	—	—
	Postintervention	4.26 (1.34)	4.26 (1.09)	0.15 (−0.47 to 0.89)	0.01
	12‐week follow‐up	4.40 (1.12)	4.40 (1.05)	0.21 (−0.59 to 0.90)	0.01
Overall participation: community involvement Measured by PEM‐CY Unit: score points 0–7	Baseline	4.31 (0.67)	4.47 (0.69)	—	—
	Postintervention	4.52 (0.77)	4.63 (0.68)	−0.15 (−0.45 to 0.35)	0.15
	12‐week follow‐up	4.60 (0.63)	4.60 (0.68)	−0.05 (−0.60 to 0.28)	0.01
Overall participation: community desire for change Measured by PEM‐CY Unit: percentage	Baseline	49.33 (28.65)	62.63 (28.75)	—	—
	Postintervention	64.21 (25.45)	56.31 (30.40)	3.70 (−9.79 to 17.20)	0.28
	12‐week follow‐up	68.84 (24.87)	44.66 (29.97)	2.01 (−11.48 to 15.52)	0.29
Overall participation: community number of activities Measured by PEM‐CY Unit: percentage	Baseline	5.52 (2.31)	5.57 (2.09)	—	—
	Postintervention	6.15 (2.00)	5.94 (2.06)	0.26 (−0.74 to 1.46)	0.09
	12‐week follow‐up	5.86 (2.16)	6.46 (2.16)	−0.33 (−1.43 to 0.75)	0.27

Abbreviations: 10X5ST, 10 metres per 5 seconds sprint test; CI, confidence intervals; COPM, Canadian Occupational Performance Measure; Kids‐Mini‐BESTest, Kids Mini Balance Evaluation Systems Test; LPA, light physical activity; MPST, Muscle Power Sprint Test; MVPA, moderate to vigorous physical activity; PEM‐CY, Participation and Environment Measure for Children and Youth; PLP‐Quest, Physical Literacy Profile‐Questionnaire; SD, standard deviation; TGMD‐2, Test of Gross Motor Development, Second Edition.

## DISCUSSION

The main aim of this randomized controlled trial was to evaluate the effectiveness of Sports Stars Brazil for children with CP in GMFCS levels I and II. Compared with the usual physical therapy group who received sessions once or twice per week, this practitioner‐led, peer‐group, modified sports intervention was superior in improving leisure‐time physical activity participation goals related to attendance and involvement, as well as motor skills performance. Additionally, Sports Stars Brazil was similar to usual physical therapy in enhancing motor skills capacity and physical literacy in this group of children. Our study contributes to the growing evidence supporting modified sports as a rehabilitation intervention for children with CP, particularly in low‐income and middle‐income countries such as Brazil. Below we reflect on this study's results through the components of modified sports' treatment.

Similar to the original Sports Stars programme in Australia, the greater COPM scores for gains in leisure‐time physical activity participation occurred postintervention and were maintained at follow‐up.^13^ In this present study, we believe that the differences in participation goals after the conclusion of the intervention—attendance and involvement—favouring Sports Stars Brazil might be because this intervention included active ingredients related to all domains of physical literacy (physical, cognitive, social, and psychological), building skills for the children's participation goals. In contrast, children in the usual physical therapy group might have engaged primarily with the physical domain (Table S1). According to previous reviews evaluating the effectiveness of participation‐focused interventions, contextual factors—including children's personal characteristics—must be considered when aiming to enhance participation across different settings.^36^ In the context of leisure‐time physical activity, these personal factors are closely linked to the domains of physical literacy.^8^ Our findings reinforce the notion that improving attendance and involvement in physical activities requires a comprehensive understanding of a child's physical literacy. We believe that Sports Stars Brazil promoted children's physical literacy skills for further improvement in their leisure‐time physical activity participation goals (i.e. participation in real‐life sports or physical recreation).

Considering these results with participants' attendance and involvement goals, we believe that Sports Stars Brazil is aligned with the Family of Participation Constructs of Imms et al.,^17^ and that attendance and involvement in participation are related to intrinsic (sense of self, preferences, and activity competence) and extrinsic (environmental) factors. As seen in our previous qualitative study,^12^ the Sports Stars programme not only enhances children's activity competence by facilitating gains in their motor skills, but also builds their confidence (sense of self) and fosters interest in physical activities (preferences). These opportunities for practice in real‐life environments with peers and the social, cognitive, and psychological demands encompass most of the participation constructs, providing a favourable context for enhancing leisure‐time physical activity. We believe it is crucial to consider these ingredients to promote leisure‐time physical activity participation. Including these ingredients ensures that a modified sports intervention aligns with its definition: ‘Modified sports programs are offered to engage children in play activities designed, among other things, to develop fundamental motor skills and sport‐specific skills for future participation’.^6^

Even though the usual therapy group received considerably more intervention (12–24 hours of therapy vs. 8 hours), Sports Stars Brazil was more effective in promoting children's motor performance, as measured by the COPM performance goals (i.e. in everyday environments). Also, both groups showed similar postintervention results in motor skill capacity, assessed in controlled settings by the Challenge and TGMD‐2 tests. Although the interventions provided to the usual physical therapy group were not formally standardized, parents and caregivers reported that children in this group engaged in individual task practice of gross motor skills, strength, and balance training in a clinical setting. We hypothesize that therapists selected active ingredients in these interventions to improve children's motor skills directly or indirectly.^37^ This might explain the key difference between the groups in motor performance and the similarity in motor capacity. While both interventions targeted motor capacity through a shared active ingredient—structured and repeated task practice—the additional ingredients required to improve motor performance, such as practice in real‐life environments, were probably present only in the Sports Stars Brazil group. As Jackman et al. emphasized, practising in real‐life contexts is essential for children with CP to transfer gains into everyday life.^38^ Sports Stars might be a cost‐effective choice of intervention to promote motor skills performance and capacity because it can produce more similar results than usual physical therapy in lower dosage parameters.

Sports Stars Brazil was not effective in improving physical activity levels, body functions, and overall participation (as measured by the Participation and Environmental Measure for Children and Youth) in the sample, immediately after, or 12 weeks after, intervention. We hypothesize that changes in body function outcomes after modified sports interventions may be achieved by programmes with higher dosage and longer duration (to effectively produce musculoskeletal changes), as seen in other modified sports studies for children with CP.^39^, ^40^ Although children participated more in physical activities after Sports Stars (according to the improvements in COPM participation goals), the magnitude of change for this outcome was small and these gains were not sufficient to promote broader lifestyle changes to decrease sedentary behaviour, immediately postintervention. As stated by Reedman et al., interventions that include behavioural change and motivational aspects are likely to better address physical activity levels in individuals with CP.^41^ Future modified sports programmes combined with strategies to improve health behaviour and motivation might achieve better outcomes. Furthermore, the lack of changes in overall participation strengthens previous findings that interventions to improve participation are not likely to produce general gains in home, school, and community contexts.^8^, ^36^

It is important to highlight that only after 12 weeks of follow‐up did children in the Sports Stars Brazil group show greater improvements in mean time spent in moderate to vigorous physical activity per week and in motor skill capacity measured by TGMD‐2, especially on object control. We believe these outcomes are indirect gains of the Sports Stars intervention. By participating in the programme, children increased their attendance and involvement in physical activities during sports and recreational sessions. As a result, after 12 weeks, they were more physically active on weekdays and had more opportunity to practise their motor skills in mainstream community physical activity programmes, improving their movement patterns when performing motor skills.

Considering the above, we hypothesize that Sports Stars Brazil might be a more economically viable option for stakeholders, particularly in low‐income and middle‐income countries such as Brazil. Compared with traditional one‐to‐one physical therapy for ambulant children with CP, this peer‐group, low‐dosage, and low‐cost intervention demonstrated superior outcomes in participation and motor performance, while achieving comparable results in motor capacity. The Sports Stars intervention required less total time (once a week, totalling 8 hours over 2 months) than usual physical therapy (typically twice a week, totalling 12–24 hours over the same period). Additionally, it can be delivered with a lower therapist‐to‐child ratio (one to four therapists for four to eight children simultaneously), unlike traditional therapy, which is commonly provided in a one‐to‐one format. This intervention format could facilitate the provision of physical therapy and habilitation services for ambulant children with CP, especially in public health systems. Future economic analysis studies could confirm this hypothesis.

Future studies could evaluate the appropriate dosage parameters of modified sports interventions to achieve secondary body function outcomes important for children with CP. In addition, considering that participation is highly influenced by one's context, we hypothesize that future studies combining modified sports interventions with context‐focused approaches might increase children's physical activity levels, reduce sedentary behaviour, and remove environmental barriers to leisure‐time physical activity participation. A study is currently underway to evaluate the feasibility of combining Sports Stars Brazil with a context‐focused therapy.^42^

This study has some limitations worth noting. One is that, although our sample size was appropriate for our primary outcome, it was sufficiently powered (power of 22%–64%) to investigate differences in some of our secondary outcomes such as physical literacy and the motor capacity tests. Future studies with a larger sample size are needed to further investigate the effectiveness of modified sports interventions in this study's secondary outcomes. Also, this study compared Sports Stars with general usual physical therapy care; future studies should also compare this intervention with specific controlled interventions (e.g. task‐specific training). In addition, our study failed to collect actual data about the usual physical therapy group, because we could not reach children's therapists, relying instead on parents' and caregivers' recalls. Information about the treatment and adherence of the control group should be better collected in future studies. Another potential limitation is the possibility of selection bias due to participant attrition during the follow‐up period, because we lost some participants in our secondary outcomes. Lastly, owing to the lack of interpretability studies of most of the selected instruments and questionnaires, analysis of the proportion of patients who achieved minimal clinically important differences was not possible.

## CONCLUSION

Sports Stars Brazil was effective in improving leisure‐time physical activity participation goals related to attendance and involvement among ambulant children with CP. This intervention enhanced participants' motor performance in fundamental motor skills, yielding results comparable to usual physical activity programmes in terms of motor skills capacity and physical literacy. No improvements were observed in physical activity levels, body functions, or overall participation after the Sports Stars Brazil programme. This modified sports intervention is an effective and low‐cost approach that has the potential for implementation in clinical practice, especially in low‐income and middle‐income countries.

## CONFLICT OF INTEREST STATEMENT

The authors declare no conflicts of interest.

## Supporting information


**Figure S1**: CONSORT diagram.

**Table S1**: Usual therapy group: Physical therapy information according to parents and caregivers.

**Table S2**: Changes over the time for Sports Stars Brazil and usual physical therapy groups.

## Data Availability

Data sharing is not applicable to this article as no new data were created or analyzed in this study.
