# Supplementary material for: Modified sports intervention for improving participation goals and activity competence in ambulant children with cerebral palsy: A randomized controlled trial
Source: Dev Med Child Neurol. 2025 Jul 3;68(1):128–41. doi: 10.1111/dmcn.16393 (PMC12683307; doi:10.1111/dmcn.16393)
Supplement: Supplementary file 1 — Figure S1: CONSORT diagram. [file DMCN-68-128-s003.pdf]

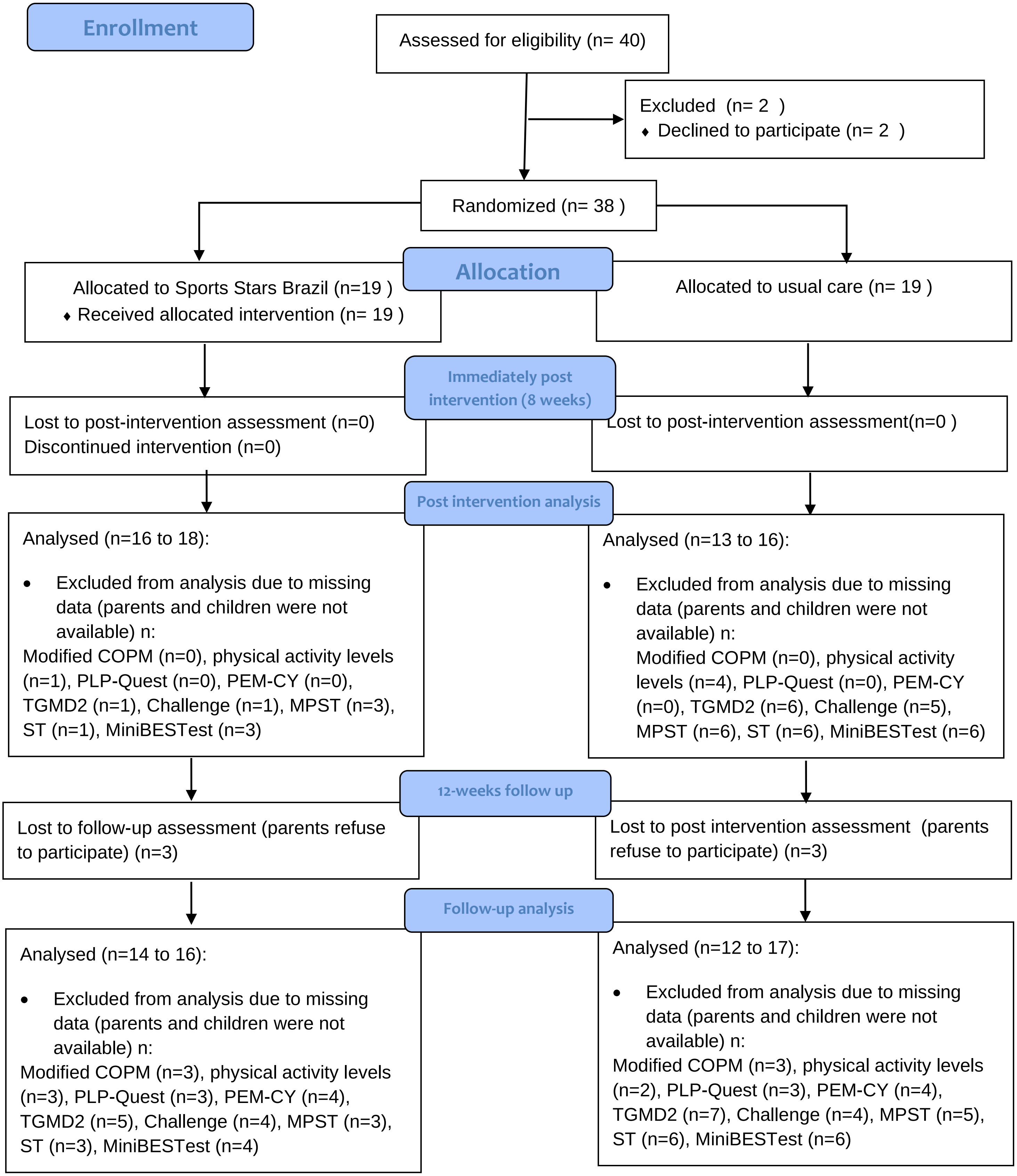

*Legend: COPM: Canadian Occupational Performance Measure; MiniBESTest: Mini Balance Evaluation Systems Test; MPST: Muscle Power Sprint Test; PEM-CY: Participation and Environment Measure for Children and Youth; PLP-Quest: Physical Literacy Profile Questionnaire; ST: 10x5 Sprint Test; TGMD-2: Test of Gross Motor Development-2
