# Supplementary material for: Modified sports intervention for improving participation goals and activity competence in ambulant children with cerebral palsy: A randomized controlled trial
Source: Dev Med Child Neurol. 2025 Jul 3;68(1):128–41. doi: 10.1111/dmcn.16393 (PMC12683307; doi:10.1111/dmcn.16393)
Supplement: Supplementary file 2 — Table S1: Usual therapy group: Physical therapy information according to parents and caregivers. [file DMCN-68-128-s002.docx]

| Table S1. Usual therapy group: Physical therapy information according to parents and caregivers | | | | | | |
| --- | --- | --- | --- | --- | --- | --- |
| Child’s ID | **Where?** | **How Much?** | **How long?** | **Setting** | **Exercises and interventions**  **(according to parents and caregivers)** | **Have they participated in the most of the sessions (>50%) over the 8 weeks?** |
| 1 | Home-based | 2 | 45 minutes | Individual/ Clinical environment | Balance exercises, strength training | Yes |
| 2 | Public service | 1 | 45 minutes | Individual/ Clinical environment | Strength training,  Gross motor activities training | Yes |
| 3 | Public service | 1 | 45 minutes | Individual/ Clinical environment | Not reported | Yes |
| 4 | Public service | 1 | 45 minutes | Individual/ Clinical environment | Not reported | No (family difficulties in attending to the sections) |
| 5 | Public service | 1 | 45 minutes | Individual/ Clinical environment | Not reported | No |
| 6 | Private service | 1 | 60 minutes | Individual/ Clinical environment | Running training  Coordination and dynamic balance exercises | Yes |
| 7 | Public service | 1 | 45 minutes | Individual/ Clinical environment | Not reported | No (services unavailability) |
| 8 | Private service | 2 | 45 minutes | Individual/ Clinical environment | Body weight support treadmill training, strength training | Yes |
| 9 | Public service | 2 | 30 minutes | Individual/ Clinical environment | Mobility exercises and strength training, running training on the treadmill | Yes |
| 10 | Public service | 2 | 45 minutes | Individual/ Clinical environment | Not reported | Yes |
| 11 | Public service | 1 | 45 minutes | Individual/ Clinical environment | Strength training | Yes |
| 12 | Public service | 2 | 45 minutes | Individual/ Clinical environment | Joint mobility exercises, strength training, gross motor training | Yes |
| 13 | Public service | 2 | 45 minutes | Individual/ Clinical environment | Joint mobility exercises, gross motor activities training | Yes |
| 14 | Public service | 2 | 45 minutes | Individual/ Clinical environment | Balance exercises, treadmill training  Gross motor activities training | Yes |
| 15 | Public service | 3 | 45 minutes | Individual/ Clinical environment | Not reported | Yes |
| 16 | Private service | 1 | 45 minutes | Individual/ Clinical environment | Not reported | Yes |
| 17 | Public service | 1 | 45 minutes | Individual/ Clinical environment | Strength training, circuit | Yes |
| 18 | Public service | 2 | 45 minutes | Individual/ Clinical environment | Gross motor activities training | Yes |
| 19 | Private service | 2 | 60 minutes | Individual/ Clinical environment | Gross motor activities training, game therapy, balance exercises | Yes |
