# Supplementary material for: Modified sports intervention for improving participation goals and activity competence in ambulant children with cerebral palsy: A randomized controlled trial
Source: Dev Med Child Neurol. 2025 Jul 3;68(1):128–41. doi: 10.1111/dmcn.16393 (PMC12683307; doi:10.1111/dmcn.16393)
Supplement: Supplementary file 3 — Table S2: Changes over the time for Sports Stars Brazil and usual physical therapy groups. [file DMCN-68-128-s001.docx]

| Table S2. Changes over the time for Sports Stars Brazil and Usual Physical Therapy groups | | | | | | | | | | | | | | | |
| --- | --- | --- | --- | --- | --- | --- | --- | --- | --- | --- | --- | --- | --- | --- | --- |
| Primary outcomes | | | | | | | | | | | | | | | |
|  | **Sports Stars Brazil** | | | | | | | **Usual Physical Therapy Care** | | | | | | | |
|  | **Baseline**  **Mean (SD)** | **Post intervention**  **Mean (SD)** | **Follow up**  **Mean (SD)** | **Post intervention difference** | ***d*** | **Follow up difference*** | ***d*** | **Baseline**  **Mean (SD)** | **Post intervention**  **Mean (SD)** | **Follow up**  **Mean (SD)** | **Post intervention difference*** | ***d*** | **Follow up difference*** | ***d*** |  |
| Participation involvement goal-Performance  Measured by COPM | 4.94 (1.80) | 7.15 (2.33) | 7.25 (2.48) | 2.21 | 1.72 | 2.31 | 1.06 | 5.31 (1.85) | 5.68 (2.18) | 6.75 (1.73) | 0.37 | 0.18 | 1.44 | 0.80 |  |
| Participation involvement goal-Satisfaction  Measured by COPM | 4.94 (2.50) | 7.73 (2.28) | 8.00 (2.25) | 2.79 | 1.99 | 3.06 | 1.18 | 4.52 (2.29) | 5.21 (2.67) | 6.43 (2.36) | 0.69 | 0.27 | 1.91 | 0.82 |  |
| Participation attendance goal-Performance  Measured by COPM | 3.00 (2.47) | 5.77 (3.42) | 6.33 (3.43) | 2.77 | 0.30 | 3.33 | 1.11 | 2.33 (1.97) | 3.05 (2.53) | 3.73 (2.98) | 0.72 | 0.31 | 1.40 | 0.55 |  |
| Participation attendance goal-Satisfaction  Measured by COPM | 2.55 (2.40) | 6.16 (3.71) | 6.60 (3.52) | 3.00 | 0.48 | 3.44 | 1.34 | 3.16 (3.05) | 3.50 (3.24) | 4.26 (3.59) | 0.95 | 0.36 | 1.71 | 0.33 |  |
| Secondary outcomes | | | | | | | | | | | | | | | |
|  | **Sports Stars Brazil** | | | | | | | **Usual Physical Therapy Care** | | | | | | | |
|  | **Baseline**  **Mean (SD)** | **Post intervention**  **Mean (SD)** | **Follow up**  **Mean (SD)** | **Post intervention difference** | ***d*** | **Follow up difference*** | ***d*** | **Baseline**  **Mean (SD)** | **Post intervention**  **Mean (SD)** | **Follow up**  **Mean (SD)** | **Post intervention difference*** | ***d*** | **Follow up difference*** | ***d*** |  |
| Motor performance goal-Performance  Measured by COPM | 4.57 (1.74) | 7.21 (1.96) | 7.71 (1.82) | 2.64 | 1.42 | 3.14 | 0.64 | 5.26 1.66) | 5.68 (1.85) | 6.06 (1.87) | 0.42 | 0.23 | 1.49 | 0.47 |  |
| Motor performance goal-Satisfaction  Measured by COPM | 5.47 (2.16) | 8.31(2.10) | 8.56 (1.89) | 2.84 | 0.84 | 3.09 | 1.15 | 5.68 (2.23) | 6.05 (2.23) | 6.50(2.65) | 0.37 | 0.16 | 0.82 | 0.33 |  |
| Physical activity levels- Sedentary behavior  Measured by Accelerometer | 59.45 (13.73) | 57.29 (11.86) | 55.52 (10.97) | -2.16 | 0.16 | -3.93 | 0.31 | 54.48 (9.94) | 57.01 (8.60) | 55.73 (10.45) | 1.25 | 0.10 | 2.53 | 0.12 |  |
|  | **Sports Stars Brazil** | | | | | | | **Usual Physical Therapy Care** | | | | | | |  |
|  | **Baseline**  **Mean (SD)** | **Post intervention**  **Mean (SD)** | **Follow up**  **Mean (SD)** | **Post intervention difference** | ***d*** | **Follow up difference*** | ***d*** | **Baseline**  **Mean (SD)** | **Post intervention**  **Mean (SD)** | **Follow up**  **Mean (SD)** | **Post intervention difference*** | ***d*** | **Follow up difference*** | ***d*** |  |
| Physical activity levels- Time in LPA  Measured by Accelerometer | 30.11 (10.01) | 31.52 (8.09) | 32.19 (7.72) | 1,41 | 0.15 | 2.18 | 0.23 | 33.62 (5.70) | 32.07 (5.06) | 33.34 (8.06) | -1.55 | 0.28 | -0.28 | 0.04 |  |
| Physical activity levels- Time in MVPA  Measured by Accelerometer | 10.37 ( 4.35) | 11.17 (4.58) | 12.18 (4.78) | 0.80 | 0.17 | 1.81 | 0.39 | 11.87 ( 4.88) | 10.86 (4.11) | 10.53 (3.46) | -0.70 | 0.22 | -1.34 | 0.31 |  |
| Physical activity levels- Mean MVPA/Week  Measured by Accelerometer | 65.63(28.68) | 64.29 (32.81) | 68.56 (31.32) | -1.34 | 0.04 | 2.93 | 1.09 | 69.05(20.64) | 61.53 (21.89) | 51.76 (20.52) | -7.52 | 0.35 | -17,29 | 0.84 |  |
| Physical literacy  Measured by PLP-Quest | 69.94 (15.79) | 81.69 (15.02) | 79.05 (19.12) | 11.75 | 1.08 | 9.11 | 0.53 | 70.45 (17.29) | 75.43 (15.98) | 72.05 (20.99) | 4.98 | 0.29 | 1.60 | 0.08 |  |
| Motor skills locomotor capacity  Measured by TGMD2 | 29.15 (10.34) | 32.33 (9.57) | 34.66 (9.16) | 3.18 | 0.31 | 5.51 | 0.56 | 31.22 (9.34) | 37.38 (9.81) | 32.16 (9.90) | 6.16 | 0.64 | 0.94 | 0.09 |  |
| Motor skills object control capacity  Measured by TGMD2 | 28.40 (10.67) | 34.55 (7.42) | 38.00 (5.73) | 6.15 | 0.66 | 9.60 | 1.12 | 31.23 (8.73) | 33.23 (6.98) | 33.46 (8.15) | 2.00 | 0.25 | 2.23 | 0.27 |  |
| Motor skills overall capacity  Measured by TGMD2 | 57.55 (18.72) | 66.88 (15.86) | 72.92 (13.68) | 9.33 | 0.75 | 15.37 | 0.93 | 62.47 (17.15) | 70.61 (16.00) | 65.08 (16.53) | 7.84 | 0.49 | 2.61 | 0.17 |  |
| Motor skills capacity  Measured by Challenge test | 35.84 (20.10) | 41.40 (21.57) | 42.72 (19.10) | 5.56 | 0.26 | 6.88 | 0.35 | 40.55 (21.23) | 47.57 (24.67) | 42.43 (24.23) | 7.02 | 0.10 | 1.88 | 0.08 |  |
| Balance  Measured by Kids Mini BESTest | 26.25 (4.20) | 25.56 (6.98) | 26.73 (3.57) | -0.69 | 0.11 | 0.48 | 0.12 | 27.52 (2.83) | 28.38 (2.46) | 27.23 (3.39) | 0.86 | 0.32 | -0.29 | 0.09 |  |
| Muscle Power- Mean  Measured by MPST | 57.55 (18.72) | 75.23 (41.96) | 73.41 (40.56) | -6.21 | 0.14 | 4.39 | 0.09 | 62.47 (17.15) | 79.19 (66.57) | 56.16 (42.43) | 1.76 | 0.24 | -21.24 | 0.01 |  |
| Muscle Power- Peak  Measured by MPST | 81.55 (47.61) | 93.33 (51.68) | 91.36 (44.62) | 11.78 | 0.52 | 9.81 | 0.18 | 95.32 (64.17) | 94.09 (72.84) | 79.72 (59.98) | -1.23 | 0.35 | -15.60 | 0.25 |  |
| Agility  Measured by 10X5 ST |  | 39.16 (19.79) | 35.37 (6.26) | 2.67 | 0.16 | -1,12 | 0.11 | 36.03 (11.95) | 35.26 (8.68) | 37.20 (7.95) | -0.77 | 0.07 | 1.17 | 0.11 |  |
|  | **Sports Stars Brazil** | | | | | | | **Usual Physical Therapy Care** | | | | | | |  |
|  | **Baseline**  **Mean (SD)** | **Post intervention**  **Mean (SD)** | **Follow up**  **Mean (SD)** | **Post intervention difference** | ***d*** | **Follow up difference*** | ***d*** | **Baseline**  **Mean (SD)** | **Post intervention**  **Mean (SD)** | **Follow up**  **Mean (SD)** | **Post intervention difference*** | ***d*** | **Follow up difference*** | ***d*** |  |
| Overall participation- School frequency  Measured by PEM-CY | 5.36 (1.53) | 5.26 (1.28) | 5.33 (1.23) | -0.10 | 0.09 | -0.03 | 0.02 | 5.15 (1.80) | 4.84 (1.64) | 5.26 (1.38) | -0.31 | 0.18 | 0.11 | 0.06 |  |
| Overall participation- School involvement  Measured by PEM-CY | 4.42 (0.76) | 4.47 (0.61) | 4.60 (0.73) | 0.05 | 0.63 | 0.18 | 0.24 | 3.89 (1.24) | 4.15 (1.21) | 4.33 (0.89) | 0.26 | 0.21 | 0.44 | 0.40 |  |
| Overall participation- School desire for change  Measured by PEM-CY | 64.21 (34.36) | 55.78 (35.63) | 54.66 (34.19) | -8.43 | 0.24 | -9.55 | 0.27 | 71.57 (37.30) | 63.15 (34.80) | 54.66 (26.69) | -16.91 | 0.23 | -16.91 | 0.52 |  |
| Overall participation- School number of activities  Measured by PEM-CY | 3.00 (1.41) | 3.94 (1.12) | 3.60 (1.24) | 0.37 | 0.73 | 0.03 | 0.45 | 3.57 (1.12) | 3.52 (1.42) | 3,60 (1.40) | 0.52 | 0.03 | 0.60 | 0.02 |  |
| Overall participation- Community frequency  Measured by PEM-CY | 4.42 (1.26) | 4.47 (1.34) | 4.60 (1.12) | 0.05 | 0.03 | 0.18 | 0.32 | 4.42 (1.16) | 4.26 (1.09) | 4.40 (1.05) | -0.16 | 0.14 | -0.02 | 0.01 |  |
| Overall participation- Community involvement  Measured by PEM-CY | 4.31 (0.67) | 4.52 (0.77) | 4.60 (0.63) | 0.21 | 0.29 | 0.29 | 0.29 | 4.47 (0.69) | 4.63 (0.68) | 4.60 (0.63) | 0.16 | 0.23 | 0.13 | 0.19 |  |
| Overall participation- Community desire for change Measured by PEM-CY | 49.33 (28.65) | 64.21(25.45) | 68.84(24.87) | 14.88 | 0.59 | 19.51 | 0.72 | 44.66(28.75) | 56.31 (30.40) | 62.63 (29.97) | 11.65 | 0.39 | 17.97 | 0.61 |  |
| Overall participation- Community number of activities  Measured by PEM-CY | 5.52 (2.31) | 6.15 (2.00) | 5.86 (2.26) | 0.63 | 0.29 | 0.34 | 0.14 | 5.57 (2.09) | 5.94 (2.06) | 6.46 (2.16) | 0.37 | 0.17 | 0.89 | 0.41 |  |
| Legend: 10X5ST- 10 meters per 5 seconds Sprint Test, CI- Confidence intervals COPM- Canadian Occupational Performance Measure; LPA- Light Physical Activity, MVPA- Moderate to Vigorous Physical Activity; MPST- Muscle Power Sprint Test, PEM-CY- Participation and Environment Measure for Children and Youth; TGMD-2- Test of Gross Motor Development Second Edition; SD- Standard deviation | | | | | | | | | | | | | | | |
